# A new caimanine alligatorid from the Middle Eocene of Southwest Texas and implications for spatial and temporal shifts in Paleogene crocodyliform diversity

**DOI:** 10.7717/peerj.10665

**Published:** 2021-01-15

**Authors:** Michelle R. Stocker, Christopher A. Brochu, E. Christopher Kirk

**Affiliations:** 1 Department of Geosciences, Virginia Polytechnic Institute and State University (Virginia Tech), Blacksburg, VA, USA; 2 Department of Geological Sciences, Jackson School of Geosciences, The University of Texas at Austin, Austin, TX, USA; 3 Department of Earth and Environmental Sciences, University of Iowa, Iowa City, IA, USA; 4 Department of Anthropology, The University of Texas at Austin, Austin, TX, USA; 5 Jackson School Museum of Earth History, The University of Texas at Austin, Austin, TX, USA

**Keywords:** Cenozoic, Caimaninae, Diversity, Climate change, Fossils, Devil’s Graveyard Formation, Species distribution

## Abstract

Dramatic early Cenozoic climatic shifts resulted in faunal reorganization on a global scale. Among vertebrates, multiple groups of mammals (e.g., adapiform and omomyiform primates, mesonychids, taeniodonts, dichobunid artiodactyls) are well known from the Western Interior of North America in the warm, greenhouse conditions of the early Eocene, but a dramatic drop in the diversity of these groups, along with the introduction of more dry-tolerant taxa, occurred near the Eocene–Oligocene boundary. Crocodyliforms underwent a striking loss of diversity at this time as well. Pre-Uintan crocodyliform assemblages in the central Western Interior are characterized by multiple taxa, whereas Chadronian assemblages are depauperate with only *Alligator prenasalis* previously known. Crocodyliform diversity through the intervening Uintan and Duchesnean is not well understood. The middle Eocene Devil’s Graveyard Formation (DGF) of southwest Texas provides new data from southern latitudes during that crucial period. A new specimen from the middle member of the DGF (late Uintan–Duchesnean) is the most complete cranial material of an alligatorid known from Paleogene deposits outside the Western Interior. We identify this specimen as a caimanine based on notched descending laminae of the pterygoids posterior to the choanae and long descending processes of the exoccipitals that are in contact with the basioccipital tubera. Unlike *Eocaiman cavernensis*, the anterior palatine process is rounded rather than quadrangular. The relationships and age of this new taxon support the hypothesis that the modern distribution of caimanines represents a contraction of a more expansive early Cenozoic distribution. We hypothesize that the range of caimanines tracked shifting warm, humid climatic conditions that contracted latitudinally toward the hothouse-icehouse transition later in the Eocene.

## Introduction

Multiple episodes of global cooling and warming events in the Cenozoic are well documented through isotopic proxies for global sea surface temperature as preserved in benthic micro-invertebrates from deep-sea sediment cores (Zachos et al., 2001; Zachos, Dickens & Zeebe, 2008) and corroborated by regional terrestrial geochemical studies (Zanazzi et al., 2007). Some of the most dramatic transitions in global climate were from an extremely warm and humid climate during the Paleocene–Eocene Thermal Maximum and the Early Eocene Climatic Optimum to a much cooler and drier climate at the end of the Eocene and into the Oligocene (Evanoff, Prothero & Lander, 1992; Zachos et al., 2001; Zanazzi et al., 2007; Woodburne, Gunnell & Stucky, 2009). A second peak in global temperature occurred in the mid-Miocene following a reduction in the extent of Antarctic ice sheets (Flower & Kennett, 1994; Zachos et al., 2001; Shevenell, Kennett & Lea, 2004; Lewis et al., 2007).

Major changes in the composition of the marine and terrestrial biota are correlated with the dramatic decrease in global temperature at the Eocene–Oligocene boundary (Berggren & Prothero, 1992; Stucky, 1992). Among terrestrial vertebrates, the general trend in North America was a decrease in the number of cosmopolitan, forest-dwelling taxa, a contraction of the ranges of higher-level clades, and an increase in regionally endemic taxa (Berggren & Prothero, 1992; Robinson et al., 2004).

Extant crocodyliform diversity is relatively low (27 recognized species), but there are over 200 species known for the clade when extinct taxa are included (see Brochu & Sumrall, 2020 and references therein). When both extinct and extant crocodyliform taxa are examined together, the clade displays a bimodal diversity distribution that roughly corresponds to the δ^18^O curve for the Cenozoic (Markwick, 1998) and reflect the bimodal changes in global temperature during this time (Mannion et al., 2015). Other factors may be driving this pattern (Jouve et al., 2017; Solórzano et al., 2019; Celis, Narváez & Ortega, 2020), but climate is nonetheless viewed as a major factor influencing crocodyliform diversity patterns over time.

The only living members of Alligatoroidea are two species in Alligatorinae (*Alligator mississippiensis* Daudin 1809 from southeastern North America and *A. sinensis* Fauvel 1879 from China) and six currently recognized Neotropical species (depending on the division of certain species complexes; Brochu, 1999; Godshalk, 2014; Escobedo-Galván et al., 2015; Uetz, Freed & Hošek, 2018; Muniz et al., 2018; Borges et al., 2018; Bittencourt et al., 2019; Roberto et al., 2020) within Caimaninae (*Paleosuchus* Gray, 1862, *Melanosuchus* Gray, 1862, and *Caiman* Spix, 1825). The earliest known members of both Alligatorinae and Caimaninae occur in Paleocene deposits in North and South America, respectively (Bona, 2007; Brochu, 2011; Pinheiro et al., 2013; Cossette & Brochu, 2018; Bona et al., 2018; Cidade, Fortier & Hsiou, 2020), but the known fossil record suggests a reversal of diversity fortune during the Cenozoic—disappearance of stem alligatorines was a primary driver in the observed drop in crocodyliform diversity after the middle Eocene, and there appears to be a sharp increase in caimanine diversity in the Neogene.

Alligatorinae is well documented in the Paleogene of Europe and North America (Stefano, 1905; Mook, 1932; Weitzel, 1935; Kälin, 1939; Gilmore, 1946; Wassersug & Hecht, 1967; Erickson, 1982; Brochu, 1999, 2000, 2003, 2004, 2010; Cossette & Brochu, 2018). Several taxa are known from the Paleogene of Asia (Martin & Lauprasert, 2010; Skutschas et al., 2014; Massonne et al., 2019). However, the group is mainly limited to North America during the Neogene, with only a handful of Asian occurrences that are referable to *Alligator* (Li & Wang, 1987; Thorbjarnarson & Wang, 2010; Claude et al., 2011; Shan, Cheng & Wu, 2013; Iijima, Takahashi & Kobayashi, 2016).

The known fossil record of Caimaninae is improving (Bona, Riff & Gasparini, 2013; Cidade, Fortier & Hsiou, 2020), but our understanding of early caimanine history is limited. The earliest known South American caimanines are of Paleocene and Eocene age, and they are incompletely preserved (Simpson, 1933, 1937; Bona, 2007; Brochu, 2011; Pinheiro et al., 2013; Bona et al., 2018). A substantial temporal gap separates those forms from Neogene taxa, some of which are well sampled but highly derived (Langston, 1965; Aguilera, Riff & Bocquetin-Villanueva, 2006; Bona, Riff & Gasparini, 2013; Hastings et al., 2013; Fortier et al., 2014; Salas-Gismondi et al., 2015; Scheyer & Delfino, 2016; Cidade et al., 2017; Souza-Filho et al., 2018). Nevertheless, there is a strong consensus from molecular and morphological data that crown caimanines are monophyletic with respect to alligatorines, and that caimanines are ultimately derived from North American ancestors (Brochu, 1999; Gatesy et al., 2003; Oaks, 2011; Pan et al., 2020).

The presence of early-branching caimanines in North America would be expected from the biogeographic distribution of living alligatorids, but enigmatic North American fossils raise questions about the simplicity of that biogeographic scenario. One extinct caimanine, *Tsoabichi greenriverensis* Brochu, 2010, is known from the Wasatchian of North America. More recently, phylogenetic analyses suggest *Bottosaurus harlani* (Von Meyer, 1832), from deposits straddling the Cretaceous–Paleogene boundary, is also a caimanine (Cossette & Brochu, 2018). However, *T. greenriverensis* and *B. harlani* appear to be derived caimanines and not at the base of the caimanine stem.

**Figure 1:**
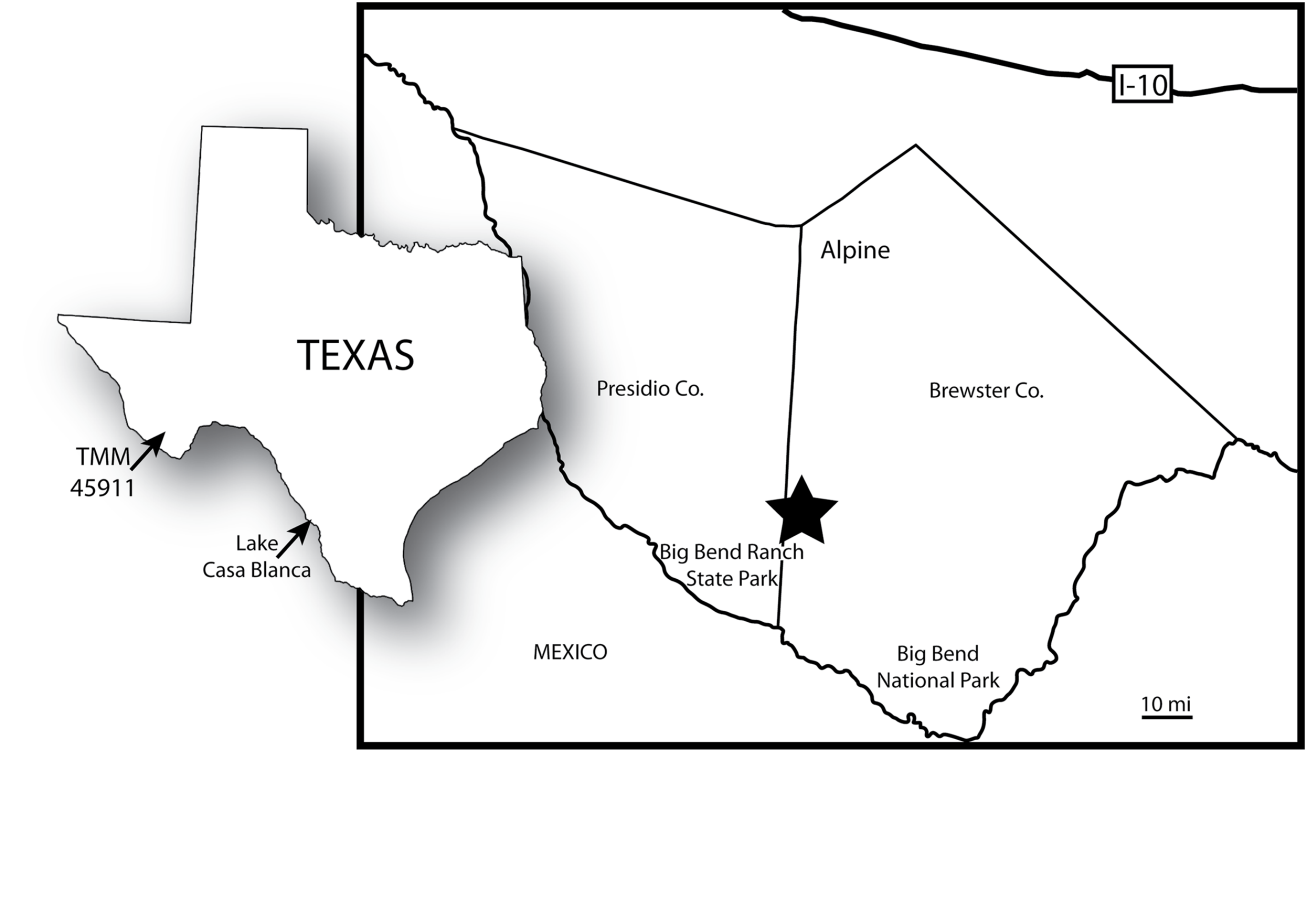
Map showing the location of TMM 45911, type locality of TMM 45911-1. At left, locations of TMM 45911 and Lake Casa Blanca. At right, map of southwest Texas. Star indicates the location of the Dalquest Desert Research Site, which includes TMM 45911.

Most of our knowledge regarding alligatorid diversity in the Eocene of North America derives from the central Western Interior (Bartels, 1983, 1984; Hutchison, 1992; Brochu, 1999, 2003, 2004, 2010). However, alligatorid occurrences from more southern fossil localities in North America hold the potential to provide key new insights into the effects of climatic deterioration following the Early Eocene Climatic Optimum. The Casa Blanca Local Fauna from near Laredo, Texas, provides a window into an estuarine faunal assemblage that existed on the western margin of the Gulf of Mexico during the late Uintan North American Land Mammal Age (NALMA) (Busbey, 1989; Westgate, 1989). That assemblage preserves planocraniids and alligatorids (Westgate, 1989), but few records document southern inland terrestrial and freshwater taxa. However, the Devil’s Graveyard Formation of Southwest Texas (Stevens, Stevens & Wilson, 1984) is a fluvially-deposited volcaniclastic sequence of fossiliferous units spanning the Uintan to Chadronian NALMAs of the Middle and Late Eocene (Stevens, Stevens & Wilson, 1984; Wilson, 1986; Campisano et al., 2014; Atwater et al., 2020). This unit preserves crocodylians and other reptiles (Brochu, 2000; Stocker & Kirk, 2011, 2016; Stocker, Brochu & Kirk, 2012), as well as a rich record of mammals that allow biostratigraphic comparisons between the terrestrial faunal assemblage of West Texas and those of the Western Interior (Fig. 1; Wilson, 1977, 1986; Runkel, 1988; Williams & Kirk, 2008; Campisano et al., 2014).

Here we provide a morphological description of a new alligatorid from the middle member (late Uintan–Duchesnean) of the Devil’s Graveyard Formation. This new taxon demonstrates a complex of derived characters shared with caimanines. As the latest occurring caimanine currently known from the Paleogene of North America, the new taxon provides important new insights into the early evolution of the Caimaninae and the past diversity of North American crocodyliforms.

## Geographic and Geologic Setting

**Figure 2:**
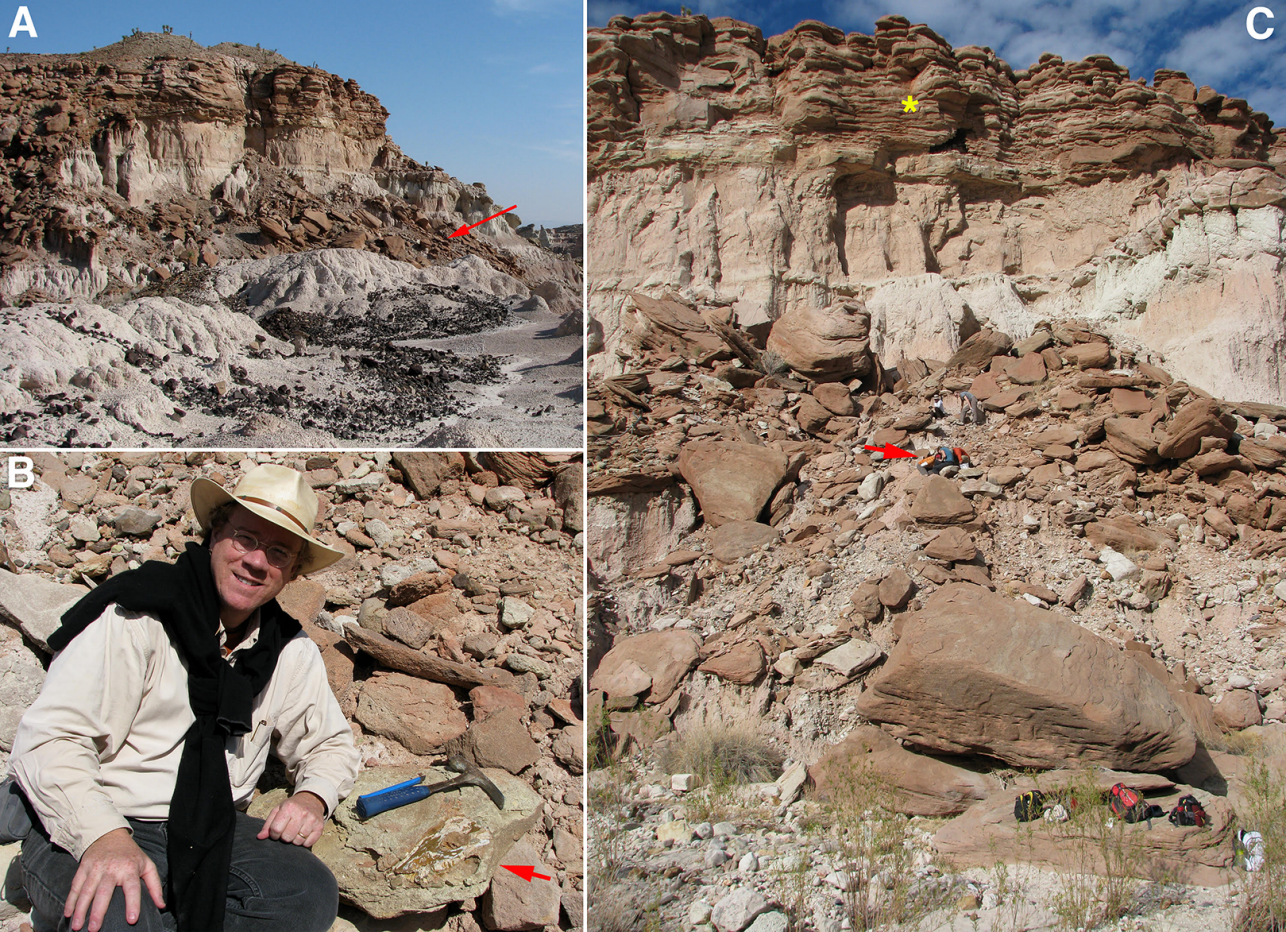
Images of the TMM 45911 fossil locality. (A) TMM 45911 (red arrow) as viewed from the west, looking east. Fossils were recovered from sandstone boulders on the talus slope of the cliff located immediately to the north. (B) TMM 45911-1 in situ shortly after its discovery in 2010, with co-discoverer Samuel Wilson. The majority of the specimen was removed from the sandstone block in a single piece by the third author in 2010 using a hammer and chisel. (C) View of TMM 45911 from the south in 2011, with the lead author (red arrow) working to remove the remainder of TMM 45911-1 (part of the quadrate). The yellow asterisk marks the channel sandstones capping the cliff to the north that are the presumptive source of the sandstone block that produced TMM 45911-1.

**Figure 3:**
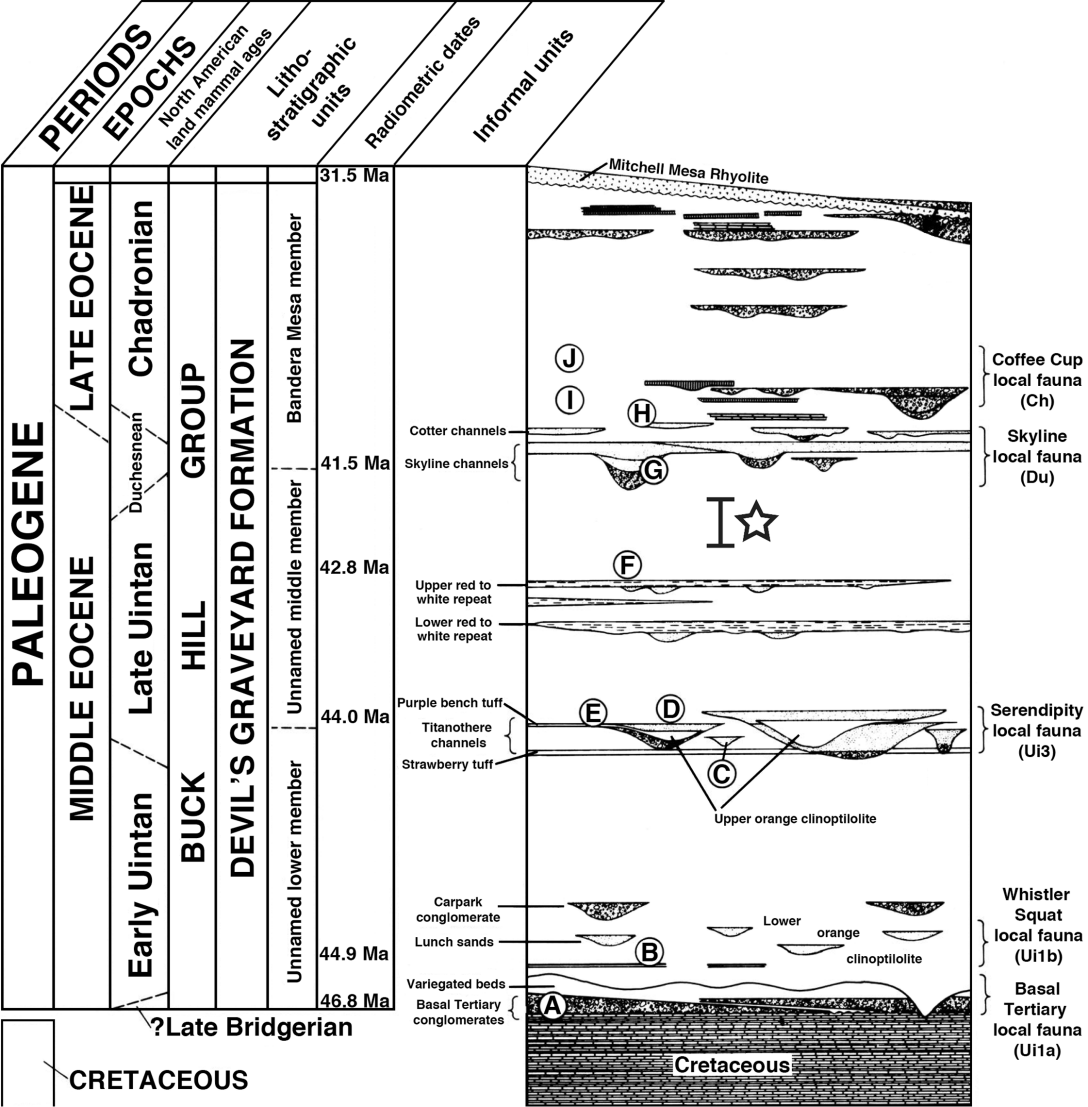
Stratigraphic column of the Devil’s Graveyard Formation, modified from Stevens, Stevens & Wilson (1984) and Wilson (1986). TMM 45911-1 was collected from a conglomeratic sandstone within the unnamed middle member of the Devil’s Graveyard Formation, above the “upper red to white repeat” and below the “Skyline channels” of Stevens, Stevens & Wilson (1984). The approximate stratigraphic provenance of TMM 45911 is indicated by the open star and bracket. Circled letters refer to key fossil localities: (A) TMM 41443 (Junction) and TMM 41444 (0.6 miles east of Junction). (B) TMM 41372 (Whistler Squat Quarry). (C) TMM 41745 (Serendipity). (D) TMM 41723 (Titanothere Hill). (E) TMM 41672 (Purple Bench). (F) TMM 46513 (Peaches). (G) TMM 41578 (Tepee Canyon). (H) TMM 41853 (Horseshoe Stone Corral). (I) TMM 41965 (Red Table). (J) TMM 41781 (Red Hill). Radiometric dates include: 46.8 Ma, basalt near Hen Egg Mountain that may overlie the fossil localities of the Basal Tertiary local fauna (Campisano et al., 2014); 44.9 Ma, best age estimate for TMM 41372 (Campisano et al., 2014); 44.0 Ma, average of 2 bracketing dates for TMM 41672 (Atwater et al., 2020); 42.8 Ma, mean age for TMM 46513 (Atwater et al., 2020); 41.5 Ma, approximate date for TMM 41578 (Campisano et al., 2019); 31.5 Ma, date of the Mitchell Mesa Rhyolite (McDowell, 1979), which intervenes between the (older) Devil’s Graveyard Formation and the (younger) Oligocene Tascotal Mesa Formation. Local faunas follow Wilson (1986), Walton (1992), and Campisano et al. (2014). Biochronological zones for local faunas follow Wilson (1986), Gunnell et al. (2009), and Campisano et al. (2014).

TMM 45911-1 was discovered in 2010 in Devil’s Graveyard Formation outcrops at Midwestern State University’s Dalquest Desert Research Site (Fig. 1), approximately 1.5 km west of the confluence of the north and south forks of Alamo de Cesario Creek. Fieldwork was approved by Midwestern State University. Detailed provenance data for this specimen are on file at the Texas Vertebrate Paleontology Collections of the Jackson School Museum of Earth History (TMM), The University of Texas at Austin. These exposures are located in the south-central portion of the Tornillo Basin, the southernmost Laramide-aged intermontane sedimentary basin in the United States (Lehman, 1991). The TMM 45911 fossil locality (“Crocolicious”) occurs within the study area of Stevens, Stevens & Wilson (1984), who originally named and mapped the Devil’s Graveyard Formation. Numerous vertebrate fossils are known from localities throughout the Devil’s Graveyard Formation (Wilson, 1986; Atwater et al., 2020), and collecting since 2005 by field parties from the University of Texas at Austin has focused primarily on the lower and middle members (Williams & Kirk, 2008; Kirk & Williams, 2011; Stocker & Kirk, 2016). TMM 45911-1 was collected from a coarse-grained, conglomeratic sandstone block that had fallen from a cliff face (Fig. 2). The channel sandstones that produced the specimen are located stratigraphically above the “upper red to white repeat” of Stevens, Stevens & Wilson (1984), a key marker bed in the unnamed middle member of the Devil’s Graveyard Formation (Fig. 3). TMM 45911 is also located statigraphically above the TMM 46513 fossil locality (“Peaches”), which has been dated to 42.8 + 1.0 − 0.4 Ma using detrital zircon U–Pb geochronological analysis (Atwater et al., 2020). TMM 45911 is located stratigraphically below the “Skyline channels” of Stevens, Stevens & Wilson (1984), which mark the base of the (upper) Bandera Mesa Member of the Devil’s Graveyard Formation. The vertebrate fauna collected from fossil localities within the Skyline channels is clearly representative of the Duchesnean NALMA (Wilson, 1986), and has been dated using ^40^Ar/^39^Ar geochronological analysis to approximately 41.5 Ma (Campisano et al., 2019). Accordingly, TMM 45911 is located within the upper half of the middle member of the Devil’s Graveyard Formation (Fig. 3), and is between approximately 42.8 and 41.5 million years old. These absolute dates suggest that TMM 45911-1 is from either the latest Uintan or earliest Duchesnean NALMA.

## Systematic Paleontology

CROCODYLIA Gmelin, 1789 sensu Clark in Benton & Clark, 1988

ALLIGATORIDAE Gray, 1844

CAIMANINAE Norell, 1988 sensu Brochu, 1999

*Chinatichampsus wilsonorum* gen. et sp. nov.

urn:lsid:zoobank.org:act:91246A7E-C9EA-4206-8A87-6205E3684989; urn:lsid:zoobank.org:act:9BA8A50C-3A37-40B2-9B35-B4D853A18890

**Holotype:** TMM 45911-1, cranium missing dorsal surface of dermal elements on left side as well as left premaxilla.

**Type Locality and Stratigraphic Occurrence:** Fossil locality TMM 45911 (“Crocolicious”). Unnamed middle member, Devil’s Graveyard Formation, late Uintan-Duchesnean (~42.8-41.5 Ma), Middle Eocene, Brewster County, Texas.

**Etymology:** *Chinati-*, for the Chinati Mountains southwest of Marfa, Texas. Pronunciation = “chee-NAH-tee”. The volcaniclastic sediments comprising the upper part of the Devil’s Graveyard Formation are partly derived from the Chinati caldera. *Champsus*, Greek for crocodile. *wilsonorum*, for Cornelia (“Nellie”) Wilson and Samuel Wilson, who discovered the holotype specimen.

**ICZN Registration and Zoobank:** The electronic version of this article in Portable Document Format (PDF) will represent a published work according to the International Commission on Zoological Nomenclature (ICZN), and hence the new names contained in the electronic version are effectively published under that Code from the electronic edition alone. This published work and the nomenclatural acts it contains have been registered in ZooBank, the online registration system for the ICZN. The ZooBank LSIDs (Life Science Identifiers) can be resolved and the associated information viewed through any standard web browser by appending the LSID to the prefix http://zoobank.org/. The LSID for this publication is: urn:lsid:zoobank.org:pub:00150C1B-9E7D-45CE-8648-12CF2188310C. The online version of this work is archived and available from the following digital repositories: PeerJ, PubMed Central and CLOCKSS.

**Diagnosis:** *Chinatichampsus wilsonorum* preserves the following unique combination of characters (characters used in this phylogenetic analysis indicated in parentheses): dorsal position of the foramen aëreum (177[1]); posterior maxillary alveoli with smaller diameters than those of the fourth and fifth maxillary alveoli; presence of prominent, notched, descending laminae of the pterygoid posterior to the choana (124[1]); presence of foramina in the medial parietal wall of the supratemporal fenestrae (154[1]); presence of supraoccipital exposure on the dorsal skull table (160[2]; shared with *Alligator prenasalis*), and presence of long descending processes of the exoccipitals that make contact with the basioccipital tubera (176[2]). It shares with *Necrosuchus* the dorsolateral position of the lateral eustachian foramina relative to the median eustachian foramen (175[0]), but differs in that the descending processes of the exoccipitals contribute to the basioccipital tubera in *Chinatichampsus*. It differs from all caimanines other than *Eocaiman cavernensis* in the presence of a posterior process of the maxilla between the lacrimal and prefrontal (128[2]), orbital margins that are flush with the skull surface (137[0]), extension of the quadratojugal to the dorsal angle of the infratemporal fenestra (145[0]; shared with alligatorines), a concavoconvex frontal-parietal suture (151[0]; shared with *Alligator mississippiensis*), the lack of an overhang of the supratemporal fenestra by the dermal skull roof elements (152[0]), and a small supraoccipital exposure on the skull table (160[0]). It can be differentiated from *Eocaiman cavernensis* by the presence of palatines that have expanded and rounded anterior margins, rather than the “irregularly quadrate” anterior margins reported for *Eocaiman cavernensis* (Simpson, 1933:3).

### Comparative Morphological Description

**Figure 4:**
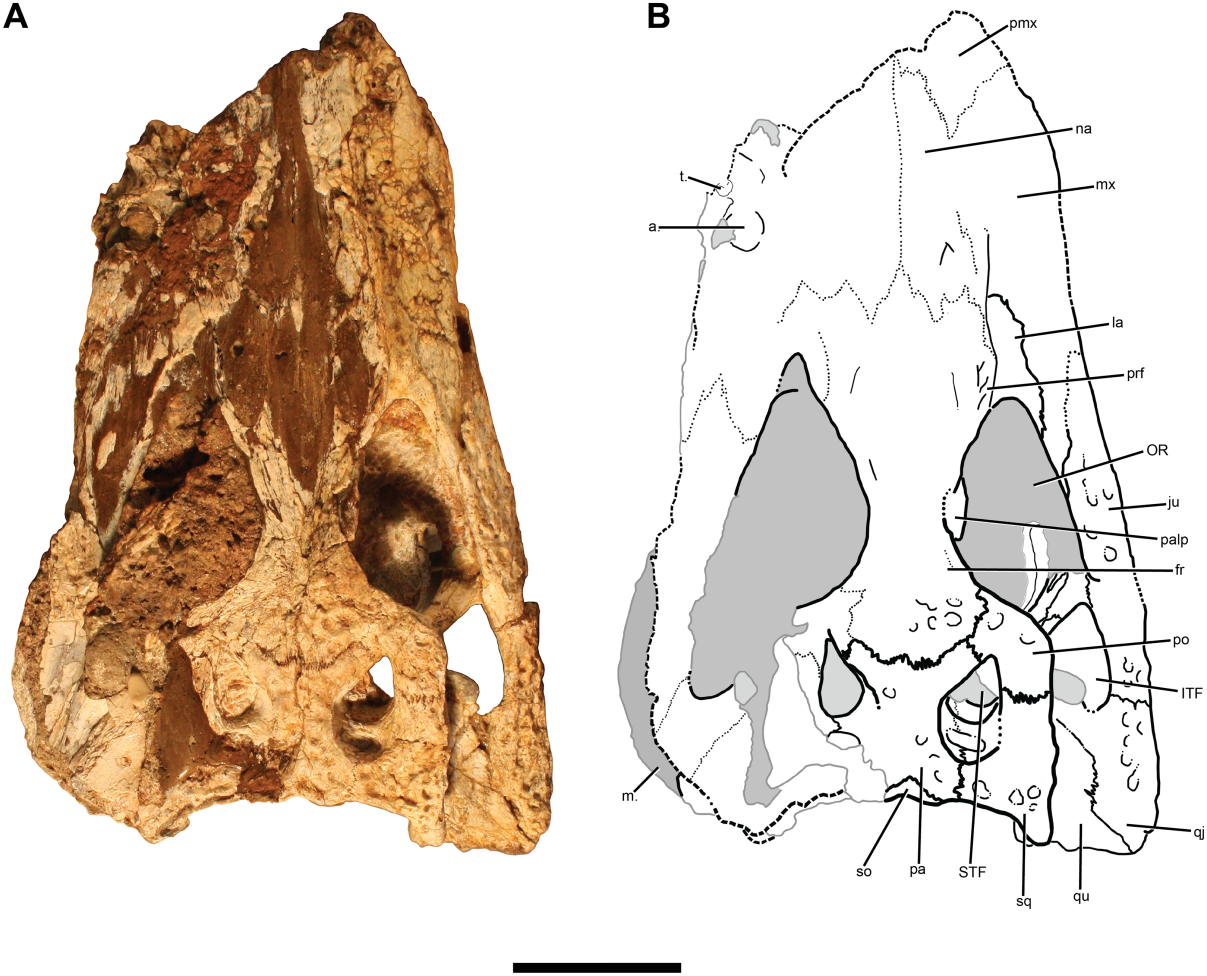
TMM 45911-1, holotype of *Chinatichampsus wilsonorum*. (A) Cranium in dorsal view. (B) Interpretive line drawing. Grey areas indicate matrix. Abbreviations: a., alveolus; fr, frontal; ITF, infratemporal fenestra; ju, jugal; la, lacrimal; m., matrix; OR, orbit; pa, parietal; palp, possible palpebral; pmx, premaxilla; po, postorbital; prf, prefrontal; qj, quadratojugal; qu, quadrate; so, supraoccipital; sq, squamosal; STF, supratemporal fenestra; t., tooth. Scale bar equals 5 cm.

**Material:** *Chinatichampsus wilsonorum* consists of a cranium that is lacking most of the dorsal surface on the left side (Fig. 4). The left premaxilla is missing, and the majority of the outer surfaces of all elements on the left side of the cranium as well as the right premaxilla, right nasal, and right prefrontal are missing. The specimen was collected in 2010 and 2011 using chisels and hammers. Mechanical preparation of this specimen was done by L. Bergwall-Herzog using a carbide needle and pneumatic airscribe. The density of the matrix surrounding the cranium made preparation of some regions difficult (i.e., the lateral portions of the braincase), and they remain unprepared. In some regions of the cranium that have eroded external surfaces, matrix was left in place either as support for the thin remaining bone or to preserve internal molds of pneumatic chambers and cranial passages. Any repair and consolidation of the bone was done with cyanoacrylate (Krazy Glue).

**Ontogenetic Status:** Though no postcrania were recovered to independently test the ontogenetic status of TMM 45911-1 through the degree of neurocentral or scapulocoracoid suture closure (Brochu, 1996), we hypothesize that it is a morphologically mature individual. In all extant crocodylians, all ontogenetic changes that would impact character state coding take place within the first one or two years of life (Brochu, 1997). TMM 45911-1 is beyond the size range at which most morphological variation caused by ontogenetic changes typically is observed (Iordansky, 1973), and though size and age are imperfectly correlated, this specimen represents an animal substantially older than 2 years of age because of size of the cranium and the relative proportions of cranial fenestrae. Therefore, the description of this specimen and any comparisons are assumed to be unaffected by ontogenetic issues.

**Figure 5:**
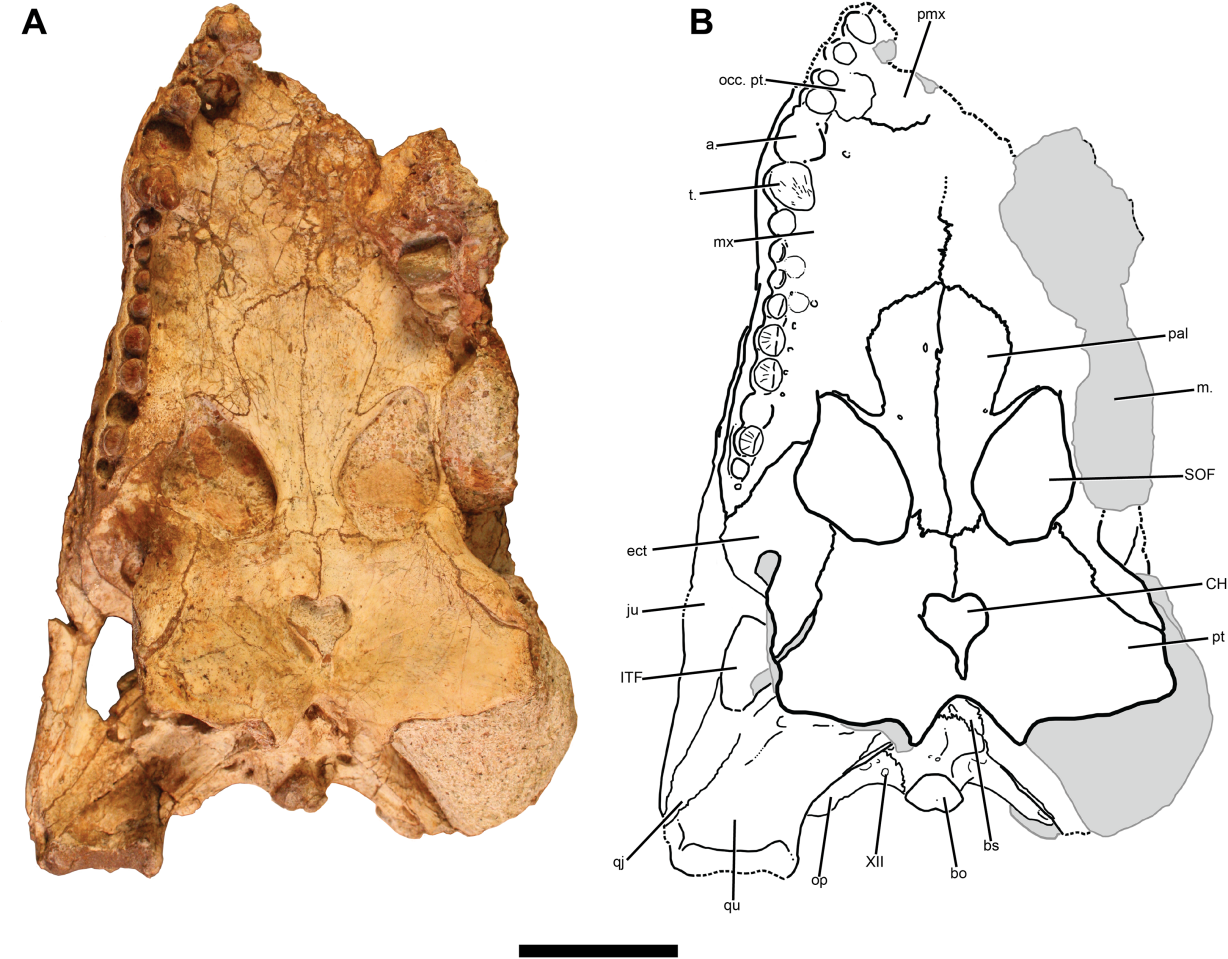
TMM 45911-1, holotype of *Chinatichampsus wilsonorum*. (A) Cranium in ventral view. (B) Interpretive line drawing. Grey areas indicate matrix. Abbreviations: a., alveoli; bo, basioccipital; bs, basisphenoid; CH, choana; ect, ectopterygoid; ITF, infratemporal fenestra; ju, jugal; la, lacrimal; m., matrix; op, opisthotic; pal, palatine; pmx, premaxilla; po, postorbital; pt, pterygoid; qj, quadratojugal; qu, quadrate; so, supraoccipital; SOF, suborbital fenestra; sq, squamosal; t., tooth; XII, foramen for Cranial Nerve XII. Scale bar equals 5 cm.

**Premaxilla:** Nearly all of the left premaxilla is broken away, and the anterior portion of the right premaxilla also is missing (Fig. 4). Dorsally, the premaxilla forms a small shelf that extends onto the dorsolateral surface of the maxilla from a position approximately dorsal to the largest maxillary teeth. On the palatal surface, the suture between the right premaxilla and maxilla is visible trending in a nearly horizontal orientation from the midline through a large and deep occlusal pit to the lateral edge of the dentition (Fig. 5). That large occlusal pit likely accommodated the fourth dentary tooth; there is no gap between the premaxillary and maxillary dentition at the point of the occlusal pit. The anteromedial portion of the palate is crushed so no clear trace of the incisive foramen can be determined.

Two teeth are preserved in the right premaxilla, and there is a slight remnant of a third alveolus at the mesial break of the right premaxilla. These are all small and conical generally, though they increase in overall size mesially.

**Maxilla:** None of the external bony surface of the left maxilla remains. Only the medial surface of the large third or fourth left maxillary tooth and traces of the posterior maxillary alveoli are still present on the lateral surface of the skull. Remnants of matrix infillings of the accessory air cavities of the nasal passage (sensu Iordansky, 1973) and the maxillary sinus are visible medial to the dorsal exposures of the alveoli.

The right maxilla preserves most of its external surface, though the outermost surface of the bone is missing or is crushed over much of the element. Anteriorly, the suture with the premaxilla is oriented posteromedially. At the posteromedial intersection of the premaxilla-maxilla suture with the nasal is a prominent crest, which trends posteriorly through the prefrontal-lacrimal suture to the anterior margin of the orbit (Fig. 4). This crest differs from the preorbital and rostral ridges observed in some caimanines (Brochu, 1999) because it is oriented anteroposteriorly rather than anterolaterally or mediolaterally.

On the ventral surface, the deep occlusal pit for the fourth dentary tooth is anteromedial to the first and second maxillary teeth. At least two additional occlusal pits along the lingual margin of the maxillary dentition show the labial position of the premaxillary and maxillary teeth relative to the dentary tooth row. The palatal laminae of the maxillae meet at the midline in an interdigitating suture. The midline contact of the maxillae is interrupted by the anterior edge of the palatines approximately at the level of the 7th maxillary alveolus. A pointed process of the maxilla is slotted into the lateral edge of the palatine, though a lateral prong of the palatine prevents the maxilla from forming any of the anteromedial border of the suborbital fenestra. A small medial shelf of the maxilla forms the anterolateral border of the suborbital fenestra before narrowing at the level of the 12th maxillary alveolus and contacting the ectopterygoid (Fig. 5).

The largest alveoli are interpreted to be the third and fourth maxillary alveoli. A large conical tooth remains in the fourth alveolus, has carinae on both the anterior and posterior faces of the tooth, and is more rounded and expanded labially than lingually. There are 13–15 maxillary teeth; what may be the posteriormost alveolus is less than half the diameter of the penultimate alveolus. The number of maxillary tooth positions is ambiguous because of two unresolved issues—the position of the maxillary-premaxillary suture and the nature of a small hole at the posterior end of the maxillary tooth row. If we assume the largest preserved alveolus (Fig. 5) is the fourth—as would be typical for any alligatorid—and that the hole is an alveolus, then there would be 15 maxillary teeth. But if we shift the maxillary-premaxillary suture posteriorly and assume the next largest alveolus is the fourth (Fig. 5) and that the hole is a pathological or taphonomic feature, then there are 13 maxillary alveoli. Moreover, although our default assumption for a fossil alligatorid would be a large fourth alveolus, the Cretaceous alligatoroid *Brachychampsa* has an enlarged fifth alveolus. Resolution of this issue requires additional specimens clarifying sutural positions and the number of distal alveoli. Alveolar diameter shows a bimodal mesiodistal distribution, with maxima proximate to the 4th or 5th and the 12th or 13th. The posterior maxillary teeth are rounded and slightly bulbous as is typical of globidont crocodyliforms. The posteriormost preserved tooth is nearly fungiform. However, the posterior maxillary teeth in *Chinatichampsus* are smaller than the largest anterior maxillary teeth and alveoli. All maxillary teeth in this specimen have carinae that are oriented mesiodistally on each tooth, with light striations radiating from the carina to the base of the enamel.

**Nasal:** Most of the dorsal surfaces of the relatively short and wide nasals are missing (Fig. 4). The contact between the right nasal and premaxilla is visible as an interdigitating suture trending posterolaterally from the posteromedial portion of the right naris to the posterodorsal extent of the premaxilla-maxilla suture. Along the midline of the cranium are paired molds of the grooved ventral surfaces of the nasals. A fine-grained matrix preserves traces of the interdigitating sutures between the posterior portions of the nasals with the prefrontals and frontal. The anteriorly elongated nasals appear to have been tapered slightly towards their anterior articulations with the maxillae and premaxillae. Though the anteriormost portion of the cranium is not well-preserved, there appears to have been at least a slight projection of the nasals beyond the posterior extent of the nares. Whether an internarial bar would have completely bisected the nares is unknown.

**Prefrontal:** The prefrontals are poorly preserved on the dorsal surface of the cranium. Both the left and right prefrontals are missing their dorsal surfaces, exposing fine-grained matrix molds of the prefrontal sinus and olfactory region. The left lacrimal canal is preserved in this manner. Traces of vasculature are preserved in the dorsal exposure of those molds, one of which trends anteromedially, and three of which trend anterolaterally to exit the right prefrontal dorsal to the lacrimal canal (Fig. 4). An anteroposteriorly trending crest continues posteriorly from the maxilla-nasal suture onto the prefrontal-lacrimal suture, terminating as a slight overhang dorsal to the lacrimal canal.

In lateral view at the anterior portion of the orbit, the prefrontal has a dorsal and a ventral process that surround a posteromedially directed flange of the lacrimal. The ventral surface and associated extent of the prefrontal pillar cannot currently be determined because of the matrix still remaining within the orbits and suborbital fenestrae. The state of preservation of the specimen makes the anterior extent of the prefrontal with respect to the lacrimal impossible to delineate with certainty (Fig. 4); based on what remains of the prefrontal sutures, the prefrontal could extend just slightly more anteriorly than the lacrimal. However, though the anterior extent of a deeper prong of the prefrontal is decidedly anterior to the lacrimal, the superficial portion of the prefrontal could have been shorter than the lacrimal. A small process of the maxilla inserts between the lacrimal and prefrontal.

**Figure 6:**
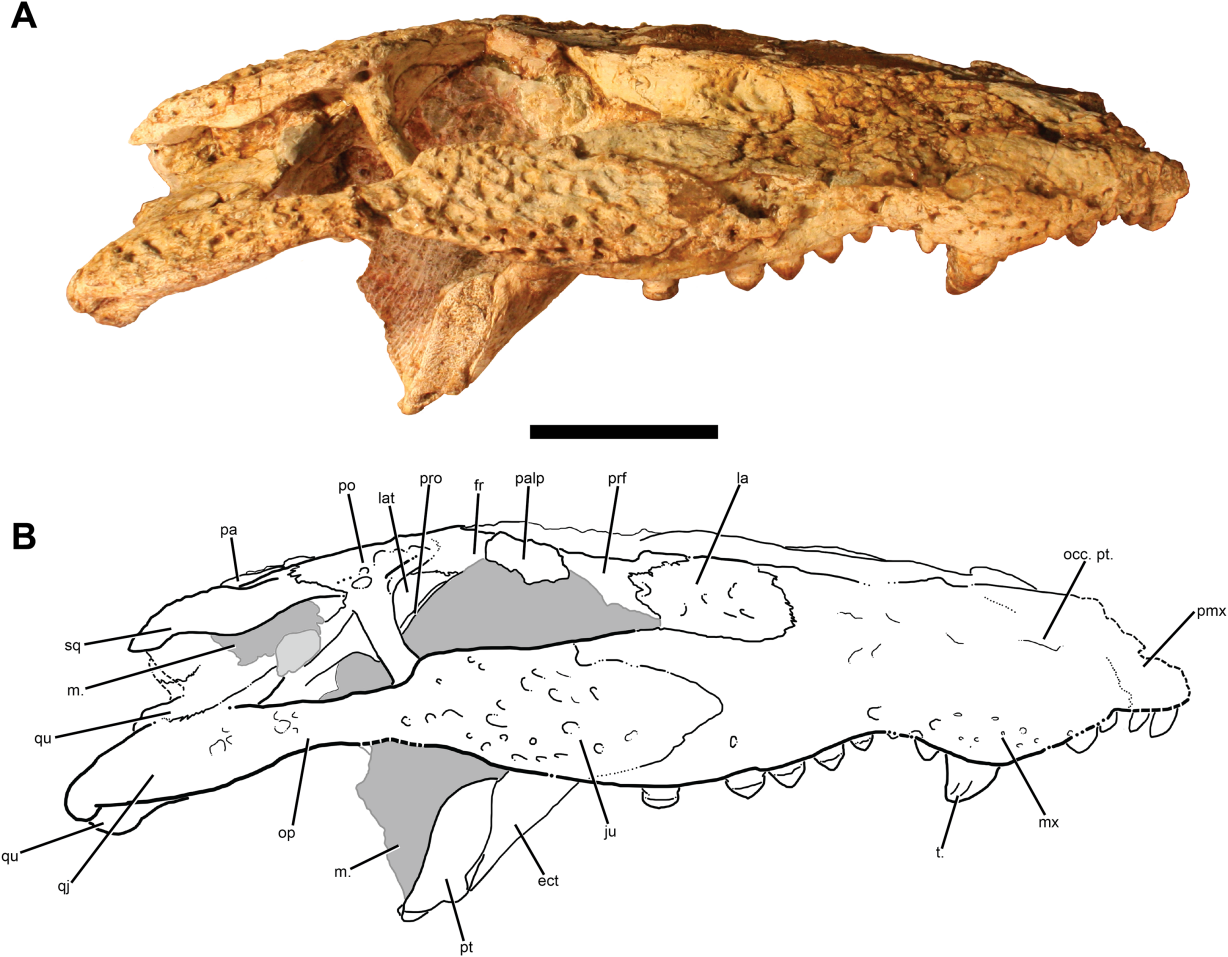
TMM 45911-1, holotype of *Chinatichampsus wilsonorum*. (A) Cranium in right lateral view. (B) Interpretive line drawing. Grey areas indicate matrix. Abbreviations: a., alveoli; fr, frontal; ITF, infratemporal fenestra; ju, jugal; la, lacrimal; lat, laterosphenoid; m., matrix; OR, orbit; pa, parietal; palp, possible palpebral; pmx, premaxilla; po, postorbital; prf, prefrontal; qj, quadratojugal; qu, quadrate; so, supraoccipital; sq, squamosal; STF, supratemporal fenestra; t., tooth. Scale bar equals 5 cm.

**Lacrimal:** The right lacrimal is well preserved. The dorsalmost edge of the lacrimal contributes to the posterior portion of the anteroposterior crest. The crest could be taphonomically emphasized because of the slight amount of crushing that occurred during preservation and erosion of the dorsal surface. However, a moderate amount of lateral concavity is present and appears to indicate that the crest would have been apparent if the cranium was complete. The anterior process of the lacrimal is oriented laterally and preserves a small area of ornamentation consisting of randomly distributed circular pits. Posteriorly the lacrimal is separated into a medial and a lateral process on either side of the anterior corner of the orbit. Within the medial process are two openings: what appears to be a blind pit anteriorly, and the entrance to the lacrimal canal posteriorly (Fig. 6). The lateral process of the lacrimal forms a short portion of the anteroventral margin of the orbit, and it appears to be separated from the jugal by a small portion of the maxilla.

**Frontal:** Much of the anterior portion of the frontal is missing, leaving behind the anterior portion of the natural endocast of the braincase. The anteriormost portion of the frontal appears to have had a deep process that extended between the nasals, as preserved by a small area of bone and the matrix-outlined suture. The frontal is narrow between the orbits. A rugose ornamentation of pits is preserved posterolaterally on the right side of the dorsal surface; the external surface of the frontal is missing on the left side. The frontal-postorbital suture interdigitates weakly, whereas the frontal-parietal suture strongly interdigitates. The frontal-parietal suture has an anteriorly concave orientation between the supratemporal fenestrae (Fig. 4). The frontal is excluded from the supratemporal fenestra by articulated processes of the parietal and postorbital. Ventrolaterally, the frontal contacts the laterosphenoid at the posteromedial margin of the orbit.

**Postorbital:** The superficial portion of the left postorbital is missing. The exposed internal surface shows the path of the canal that continues through the large lateral foramen; it is preserved as an internal mold. The right postorbital contacts the parietal along the anteromedial margin of the supratemporal fenestra, excluding the frontal from contacting the fenestra. Posteroventrally, the postorbital contacts the quadrate and quadratojugal at the dorsal angle of the infratemporal fenestra. The postorbital-jugal bar is inset from the lateral jugal surface, and the postorbital forms the anterior and medial surfaces of the bar. The capitate process of the laterosphenoid contacts the postorbital medial to the dorsal portion of the postorbital-jugal bar.

**Squamosal:** The squamosals form the posterolateral corners of the skull table. The dorsal surface of the right squamosal is ornamented with deep pits of various diameters; the area of the left squamosal preserves almost none of the original external surface but does preserve a fine-grained matrix mold preserving the shape of the posttemporal canal and the sinus that was ventral to the squamosal (Fig. 4). Within the supratemporal fenestra, a process of the squamosal ventral to the temporal canal contacts the parietal dorsal to the quadrate.

Laterally, the dorsal and ventral rims of the squamosal groove are roughly parallel (Fig. 6), and the ventral rim bears a curved ventral extension that is slightly more expanded than a similar structure in *Alligator mississippiensis* (e.g., TMM M-12608). The extent of the squamosal around the external auditory meatus currently cannot be discerned because of matrix covering most of this area. Posteriorly the squamosal overlies the paroccipital process; the posterolateral articulation of the squamosal with the paroccipital process is broken but would have continued farther posterolaterally than the posterodorsal corner of the squamosal.

**Parietal:** The dorsal surface of the parietal is missing on the left side, exposing a portion of the temporal canal that was infilled with matrix. Anteriorly, the strongly interdigitating suture with the frontal is anteriorly concave and located near the anterior margin of the supraoccipital fenestrae. At its anterolateral processes, the parietal articulates with the postorbital in a robust suture, excluding the frontal from participation in the supratemporal fenestra. The parietal is constricted mediolaterally at the level of the posterior margin of the supratemporal fenestra. The posterior portion of the parietal is expanded mediolaterally, though not as much as the anterior portion. A slight overhanging lip of the parietal forms a small concavity in the medial portion of the supratemporal fossa; two small foramina appear to perforate the parietal surface within that concavity. At the ventral surface of that concavity, the parietal appears to contact the laterosphenoid. The anteroposteriorly oriented parietal-squamosal suture is positioned at the approximate midpoint of the posterior margin of the supratemporal fenestra. A small portion of the parietal contacts the posterior margin of the skull table on the lateral sides of the supraoccipital, medial to the squamosals (Fig. 4).

**Supraoccipital:** The supraoccipital has a small triangular exposure on the skull table (Fig. 4). The posteriorly-directed processes for the attachments for *M. spinalis capitis* are visible in dorsal view, though this could be exaggerated by distortion of the palate and braincase. Small openings are present medial to those processes, and the posttemporal fenestrae are lateral to them. The posterior exposure of the supraoccipital also is triangular, and the surface bears a strong dorsoventrally-oriented ridge that extends nearly to the ventral contact with the exoccipital processes of the otooccipitals.

**Jugal:** Almost the entirety of the left jugal is missing; what remains is a thin lamina from the medial surface of the body of the jugal and a small section of external bone just anterior to the location of the postorbital-jugal bar. The right jugal, however, is well-preserved and appears to be missing only a small section of ornamentation along its anteriormost edges and a small wedge of the ventral margin just posterior to the right postorbital-jugal bar (Fig. 6). A rugose ornamentation of pits and ridges is preserved covering the lateral surface. Anteriorly, the suture between the jugal and maxilla is difficult to trace, though the jugal appears to have extended no more anteriorly than the 10th or 11th maxillary alveolus. Posteriorly, the sutural articulation with the quadratojugal is unclear, though it is easily observed as a “V”-shaped contact on the ventral margin of the cranium.

In medial view, the jugal has an extensive contribution to the posteromedial aspect of the postorbital-jugal bar (Fig. 5). A small foramen is present within the jugal just posterior to the bar, but the size of the medial jugal foramen cannot be determined.

**Quadratojugal:** The left quadratojugal is preserved as a thin, medial lamina; the suture between it and the jugal is visible just anterolateral to the posterior corner of the infratemporal fenestra and directed posterolaterally (Fig. 4). The right quadratojugal is slightly visible on the edge of the lateral condyle of the right quadrate; the contact follows the angle of the quadrate before leveling out into a horizontal and highly interdigitating suture. A narrow process forms the posterior edge of the infratemporal fenestra and contacts the postorbital and quadrate at the dorsal angle of the infratemporal fenestra. The presence of a quadratojugal spine is questionable; at most it would be small and, if present, remains covered in matrix.

**Quadrate:** Only the right quadrate is preserved completely. A small foramen aereum is located on the dorsal surface of the quadrate (Fig. 4). Contact between the squamosal and parietal appears to prevent the quadrate from entering the temporal canal in *Chinatichampsus*. Ventromedially, the attachment area for the posterior mandibular adductor musculature forms a modest crest. The lateral hemicondyle is larger than the medial hemicondyle. Details of the quadrate participation in the otic aperture and the foramen ovale are obscured by matrix.

**Pterygoid:** The robust pterygoids are roughly trapezoidal in ventral view and wider posteriorly than anteriorly. The pterygoids form the entire posterior margin of the suborbital fenestrae (Fig. 5). Their sutural articulations with the palatines are linear medially and are anterior to the posterior edge of the suborbital fenestrae at their lateral extent.

The pterygoids completely surround the choanae. The choanae project anteroventrally and have “raised” posterior edges posteriorly because of a surrounding concavity. A deep notch bisects the posterior edge. The choanal septum is recessed and not visible in lateral view.

**Palatine:** In ventral view, the palatines of *Chinatichampsus* are rounded and expanded anteriorly (Fig. 5); this appears to be a unique feature of this taxon. The lateral edges of the palatines narrow posteriorly toward the midline, so that at the level of the anterior edge of the suborbital fenestrae the palatines are narrower than at their anterior margins. Small, pointed processes of the palatines project anterolaterally around the anterior margins of the suborbital fenestrae. Posteriorly, the palatines continue to narrow nearly to the palatine-pterygoid articulation. The articulation with the pterygoids appears to be dorsoventrally deep and projects dorsally into the suborbital sinus. Additional preparation is needed to observe this more clearly, but this area is not easily accessible with preparation tools. The lateral portion of the palatine-pterygoid suture has an anterior inflection and is not at the posterior margin of the suborbital fenestra. The palatine does not contact the ectopterygoid, but is separated from it by a medial projection of the maxilla (Fig. 5).

**Ectopterygoid:** The dorsal process of the ectopterygoid has both an anterior and a posterior prong. The anterior prong articulates with the maxilla along the maxillary shelf and is excluded from the toothrow. That process forms the posterolateral margin of the suborbital fenestra. The posterior prong articulates with the medial side of the body of the jugal and sends a narrow process dorsally on the postorbital bar to articulate with the postorbital on its medial surface.

Medially, the ectopterygoid has a strong contact with the pterygoid but does not continue to the posterior edge of the pterygoid wing (Fig. 5). In ventral view, the articulation with the pterygoid bears a concavity approximately halfway along the suture, so that the ectopterygoid-pterygoid contact has a small bend.

**Figure 7:**
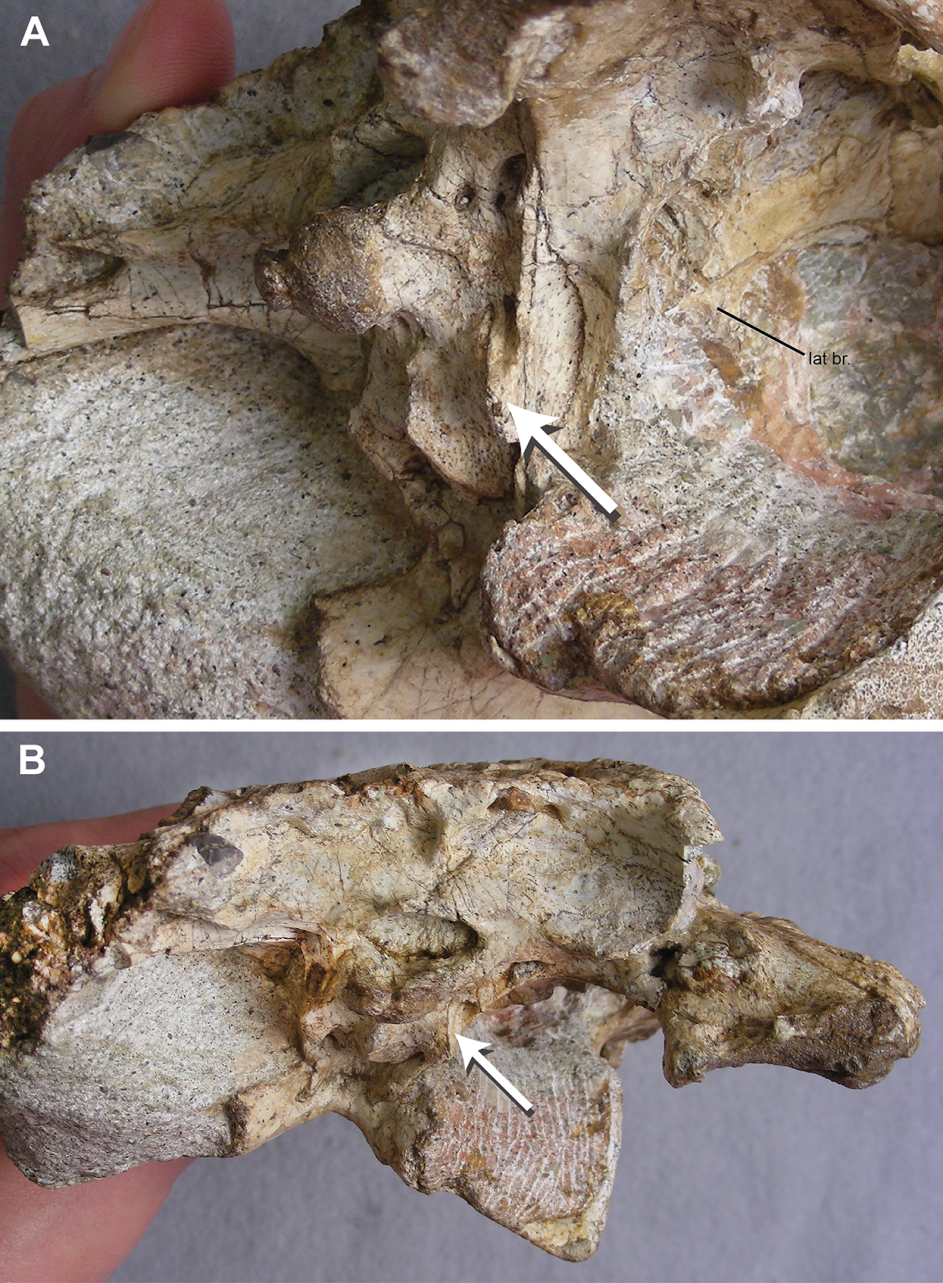
TMM 45911-1, holotype of *Chinatichampsus wilsonorum*. (A) Cranium in right posterolateral view to show the ventrally-extending exoccipital processes of the otooccipitals contributing to the basitubera (as indicated by arrow). (B) Cranium in posterior view; arrow again indicates the ventrally-extending exoccipital process of the otooccipital. Abbreviations: lat br., laterosphenoid bridge.

**Basioccipital:** The basioccipital forms the occipital condyle; the exoccipital pillars articulate on either side of a section of the dorsal surface of the basioccipital. The occipital condyle is small and slightly deflected ventrally; this could be caused by the torsion of the palate. A dorsoventrally-oriented crest is present between the basioccipital tubera, and a small foramen is present between the crest and the occipital condyle. The basioccipital tubera are wide, and bear elongated processes of the otooccipitals that nearly contribute to the tubera (Fig. 7). The medial eustachian canal opens ventrally between the basioccipital and the basisphenoid, and the lateral eustachian canals open dorsal to the medial canal.

**Basisphenoid:** There is limited exposure of the basisphenoid in either posterior or lateral views. A thin apron of the basisphenoid is exposed posteriorly, ventral to the basioccipital and appressed to the posterior surface of the pterygoids. In lateral view, the basisphenoid is exposed as a thin, “D”-shaped element between the quadrate and the otooccipital.

**Otooccipital:** The otooccipitals (exoccipitals + opisthotics sensu Evans, 2008) contact each other on the midline ventral to the supraoccipital and prevent the supraoccipital from entering the dorsal border of the foramen magnum. The foramen magnum appears slightly compressed dorsoventrally. Two foramina for cranial nerve XII exit the right exoccipital, whereas only a single foramen for cranial nerve XII is observed in the left (Fig. 5). Lateral to the foramen for cranial nerve XII, a wide and deep vagus fossa opens ventrally. The distal ends of both paroccipital processes are broken and missing, and it is unclear what their posterolateral extent would be with respect to the squamosals. The entrance to the cranioquadrate canal is visible in posteroventral view below small flanges of the paroccipital processes. Long and slender descending processes of the otooccipitals are present on the lateral surfaces of both sides of the basioccipital and contribute to enlarged lateral tubera (Fig. 7).

**Laterosphenoid and Prootic:** Although matrix obscures most of the laterosphenoid, a portion of this element is visible on the right side. The capitate process articulates with the dorsomedial corner of the postorbital-jugal bar, and the dorsal portion of the laterosphenoid continues anteriorly to contact the frontal within the orbital rim. A ventral process of the laterosphenoid forms the dorsal portion of a laterosphenoid bridge anterior to the trigeminal foramen (Fig. 7). The prootic is completely obscured by matrix on the left side, and only a possible narrow portion of the right prootic is visible through the matrix.

**Possible Palpebral:** A single flat element is preserved in the right orbit (Figs. 4 and 6). This element is in the correct location to be a palpebral, and it appears to have a bony texture to its dorsal surface. However, it is unclear whether this is actually a thin lamina of the frontal exposed on a small pebble.

### Phylogenetic Analysis

In order to assess the phylogenetic affinities of *Chinatichampsus* among other members of Alligatoridae, we coded TMM 45911-1 into the morphological character-taxon matrix of Bona et al. (2018), which is based on that of Brochu (2011). Codings for *Centenariosuchus gilmorei* Hastings et al., 2013 were modified following direct observation of specimens. We added *Bottosaurus harlani* following codings in Cossette & Brochu (2018), as well as *Diplocynodon remensis* Martin et al., 2014 based on our own observations of type and referred specimens. Codings for *Necrosuchus* are those of Brochu (2011). In the final analysis we did not include the “Uinta gator” (see below). *Notocaiman* was excluded from the analysis because of the loss of resolution its incompleteness generates. *Culebrasuchus mesoamericanus* Hastings et al., 2013 was also excluded; although some published analyses suggest *C. mesoamericanus* is an early-branching caimanine (Hastings et al., 2013; Massonne et al., 2019), others support radically different phylogenetic placements (Bona et al., 2018), and our own first-hand observations of the type material differ from those in the literature. We believe this important material requires further study before it can be included in our phylogenetic work.

Our final matrix includes 202 morphological characters (both osteological and soft-tissue) and 56 ingroup taxa. Trees were rooted using *Borealosuchus sternbergii* (Gilmore, 1910) and *Boverisuchus vorax* (Troxell, 1925) as outgroups. The matrix and character list are provided on MorphoBank under Project ID 2781 (http://morphobank.org/permalink/?P2781). *Chinatichampsus* was coded for cranial characters in this matrix only because of the lack of mandibular, postcranial, and soft tissue material in the holotype specimen. Additionally, some of the cranial characters in the matrix were either missing or uncertain for TMM 45911-1.

A maximum parsimony analysis of this matrix was executed in PAUP* 4.0b10 (Swofford, 2002) using a heuristic search, with 1,000 random addition (RA) replicates and tree bisection and reconnection (TBR) branch swapping. All multistate characters were analyzed as unordered, and all characters were equally weighted.

**Figure 8:**
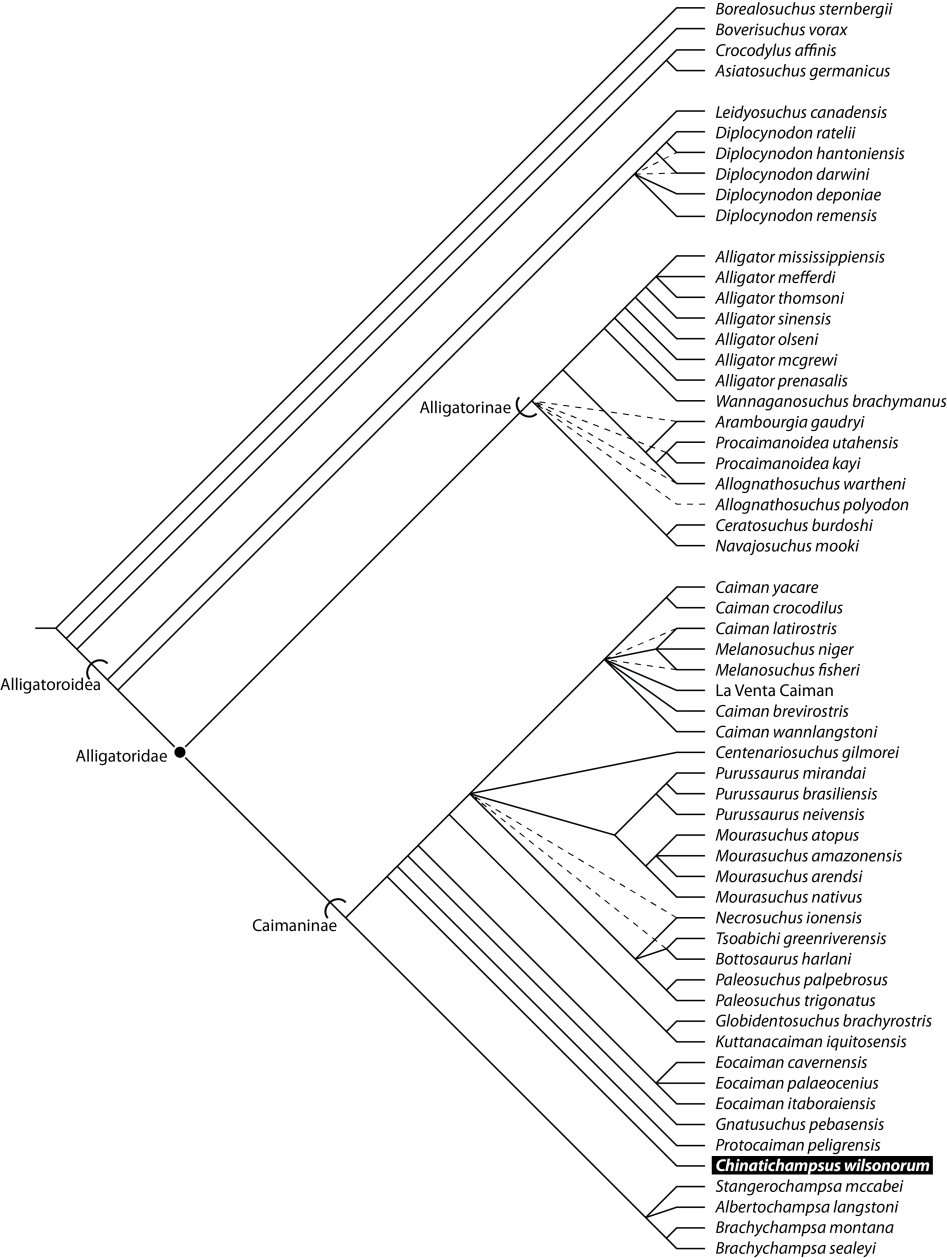
Phylogenetic relationships of Alligatoroidea including TMM 45911-1 based on the matrix of Brochu (2011) for *Necrosuchus*. *Chinatichampsus wilsonorum* (in bold) is nested within Caimaninae, branching earlier than *Eocaiman cavernensis*. Solid lines represent Adams consensus relationships, whereas dashed lines depict resolution from strict consensus.

Our analysis resulted in a set of 536 most parsimonious trees (MPTs) of tree length 413, C.I. of 0.436 without uninformative characters, and R.I. of 0.745 (Fig. 8). Relationships within this strict consensus are similar to those reported in previous analyses (Brochu, 2011; Bona et al., 2018; Cossette & Brochu, 2018) with a monophyletic Alligatoridae and a monophyletic Caimaninae. *Chinatichampsus* is recovered within Alligatoridae as an early-branching caimanine (Fig. 8).

## Discussion

*Chinatichampsus* represents a new alligatorid (Stocker & Kirk, 2011; Stocker, Brochu & Kirk, 2012). Our phylogenetic analysis recovers it as a stem caimanine, earlier-branching than *Eocaiman cavernensis* and crown caimanines (Fig. 8) and the basal-most Cenozoic caimanine. Caimaninae is unambiguously supported by the presence of an angular that does not extend dorsally beyond the anterior end of the foramen intermandibularis caudalis and has a very blunt anterior tip (65[1]; unknown in TMM 45911-1) and a naris that projects dorsally (81[1]). Ambiguous character support for Caimaninae includes a proatlas that lacks an anterior process (3[1]; unknown in TMM 45911-1), a splenial that lacks an anterior perforation for the mandibular ramus of cranial nerve V (52[1]; unknown in TMM 45911-1), an inferior process of the coronoid that remains largely on the medial surface of the mandible [57[1]; unknown in TMM 45911-1), and a maxilla with the posterior process between the lacrimal and the prefrontal (128[2]).

Support for *Chinatichampsus* + crown caimanines includes three unambiguous characters (characters 112, 124, 176). The maxilla bears a broad shelf extending into the suborbital fenestra making the lateral margin concave (112[1]), the posterior rim of the internal choana is deeply notched (124[1]), and the otooccipitals send slender processes ventrally to the basioccipital tubera (176[2]). *Chinatichampsus* does not share the following character states with later-branching caimanines: lacrimal makes broad contact with nasal with no posterior process of maxilla present (128[0]), dermal bones of skull roof overhang the rim of the supratemporal fenestra near maturity (152[1]; unambiguous), and the medial parietal wall of the supratemporal fenestra bears foramina (154[1]; unambiguous).

*Chinatichampsus wilsonorum* and *Eocaiman cavernensis* share character states that are plesiomorphic for crown Caimaninae and *Necrosuchus* (Fig. 8). Those characters include the presence of a posterior process of the maxilla between the lacrimal and prefrontal (128 2]), orbital margins that are flush with the skull surface (137[0]), extension of the quadratojugal to the dorsal angle of the infratemporal fenestra (145[0]; shared with alligatorines), a concavoconvex frontal-parietal suture (151[0]; shared with *Alligator mississippiensis*), the lack of an overhang of the supratemporal fenestra by the dermal skull roof elements (152[0]), and small, rather than large, supraoccipital exposure on the skull table (160[0]). Preorbital crests are inferred in both *Eocaiman* and in *Chinatichampsus*. The dentary dentition is medial to the premaxillary and maxillary dentition in *Eocaiman cavernensis*, and this is inferred for *Chinatichampsus* based on the occlusal pits preserved in the palate. Both taxa share approximately 14 maxillary teeth, though it is possible that there are only 13 in TMM 45911-1. The pterygoids form the entire posterior border of the suborbital foramina in both taxa. *Chinatichampsus* can be differentiated from *Eocaiman cavernensis* because of the rounded shape of the expanded anterior margins of the palatines in TMM 45911-1, rather than the quadrangular anterior margins observed in *Eocaiman cavernensis*.

TMM 45911-1 previously was hypothesized to have potential affinities with Alligatorinae (Stocker, Brochu & Kirk, 2012). That hypothesis partially was based on comparisons between TMM 45911-1 and a new Uintan alligatorine from the Uinta Basin of Utah (Brochu & Snyder, 2010; Rubin, 2020) that is currently undergoing formal description. Both taxa have a relatively short rostrum and lack enlarged posterior maxillary teeth. However, enlarged posterior maxillary teeth are shared among all other globidontans (Brochu, 1999). In both taxa the parietal and squamosal meet along the posterior wall of the supratemporal fenestra (155[2]), the quadrate foramen aëreum is on the dorsal surface of the quadrate (177[1]), and the quadrate has a small medial hemicondyle and a dorsal notch for the foramen aëreum (181[1]). TMM 45911-1 can be differentiated from the Uinta Basin taxon (Brochu & Snyder, 2010) by several characters. In the Uinta Basin taxon, the maxilla has a posterior process within the lacrimal (128[1]), but in TMM 45911-1 the maxilla has a posterior process between the lacrimal and the prefrontal (128[2]). The anterior margins of the palatines are rounded in TMM 45911-1. The caimanine synapomorphies observed in TMM 45911-1, including extensive ventral processes of the otooccipitals along the lateral edges of the basioccipital tubera, the anteroposteriorly trending ridge on the dorsal surface of rostrum, and the presence of the prominent, notched, descending lamina of the pterygoid posterior to the choana, further differentiate the two taxa.

An additional feature that was previously used to infer affinities with Alligatorinae for *Chinatichampsus wilsonorum* was the relative length of the prefrontal with respect to the lacrimal. The previous hypothesis that the prefrontal of *Chinatichampsus wilsonorum* was anteroposteriorly longer than its lacrimal was influenced by the poor preservation of that area of the holotype specimen. In TMM 45911-1, the superficial portion of the prefrontal is missing, and inferences of its length were based on the matrix infilling of the original sutural anterior edge of the element. It is unclear whether the prefrontal of *Chinatichampsus wilsonorum* would have been similar to that of alligatorines in which there is a ventral prong of the prefrontal that extends farther anteriorly than a superficial portion. If the prefrontal of TMM 45911-1 originally had a shorter superficial portion than its ventral portion, then the prefrontal likely was shorter than the lacrimal. That morphology would be in contrast to the longer prefrontal than the lacrimal in the Uinta Basin taxon and all other alligatorines. However, we conservatively treated this character as missing data for *Chinatichampsus wilsonorum* because of the poor preservation in TMM 45911-1 in this area.

Other crocodyliforms are known from lower in the Devil’s Graveyard Formation, including specimens identified as Planocraniidae (formerly Pristichampsinae), *Borealosuchus*, and Globidonta (e.g., from the Whistler Squat local fauna; Busbey, 1986; Brochu, 2000), but no specimens of crocodyloids are known. Fragments of a left dentary and splenial (TMM 41576-7) and isolated teeth (TMM 42952-113) from the Whistler Squat local fauna had represented the “southernmost known occurrence of a blunt-toothed alligatoroid in the Tertiary of North America” (Brochu, 2000:9), and TMM 45911-1 represents a more complete globidontan taxon from this latitude in the Paleogene. Additional crocodyliform material from West Texas includes specimens (identified as *Allognathosuchus*, *Boverisuchus*, Alligatorinae, and Crocodylidae; Westgate, 1989) from the Casa Blanca local fauna (Laredo Formation) of the Texas Gulf Coastal Plain. That local fauna was hypothesized to be equivalent in age to the Ui3 biochron of the Uinta Basin and to the late Uintan of West Texas (Robinson et al., 2004; Gunnell et al., 2009; Westgate, 1989, 2012). A partial skull table collected with the Casa Blanca local fauna was identified as an indeterminate alligatorine (TMM 42486-643; Westgate, 1989) and alternatively as a caimanine (Busbey, 1989), but its precise relationships were unclear because of the incompleteness of the specimen (Brochu, 2010).

Historically, isolated teeth or fragmentary specimens of globidontans from the Paleogene of Europe and North America were referred to *Allognathosuchus*, but these determinations were based on a simplified view of the early evolutionary history of Alligatoridae (Brochu, 2004). The same trend for such teeth and jaw fragments to be identified as *Allognathosuchus* applied to some previously recognized specimens from West Texas (Westgate, 1989). The posterior maxillary teeth of *Chinatichampsus wilsonorum* are bulbous and nearly spherical. However, the morphology of those teeth in combination with other cranial characters allows a more accurate systematic assessment. In *Chinatichampsus wilsonorum*, the posterior maxillary alveoli are smaller than the third and fourth/fourth and fifth maxillary alveoli, which is not the case in *Allognathosuchus* (Brochu, 2000, 2004). Based on more recent hypotheses of the evolution of globidont morphology that indicate bulbous dentition is plesiomorphic for Alligatoridae, fragmentary specimens with bulbous teeth are more conservatively assigned to Globidonta, rather than to a more exclusive taxon (Clark & Norell, 1992; Brochu, 2000, 2004). This is complicated by decreased resolution at the root of Alligatoridae, recovery of taxa previously regarded as non-crown globidontans as caimanines, and a redundancy of Globidonta and Alligatoridae in recent analyses (Cossette & Brochu, 2018; Bona et al., 2018, though they apply the name Globidonta to the node linking alligatorids and *Diplocynodon*), but enlarged distal teeth nevertheless appear to have a broad phylogenetic distribution among derived alligatoroids.

**Figure 9:**
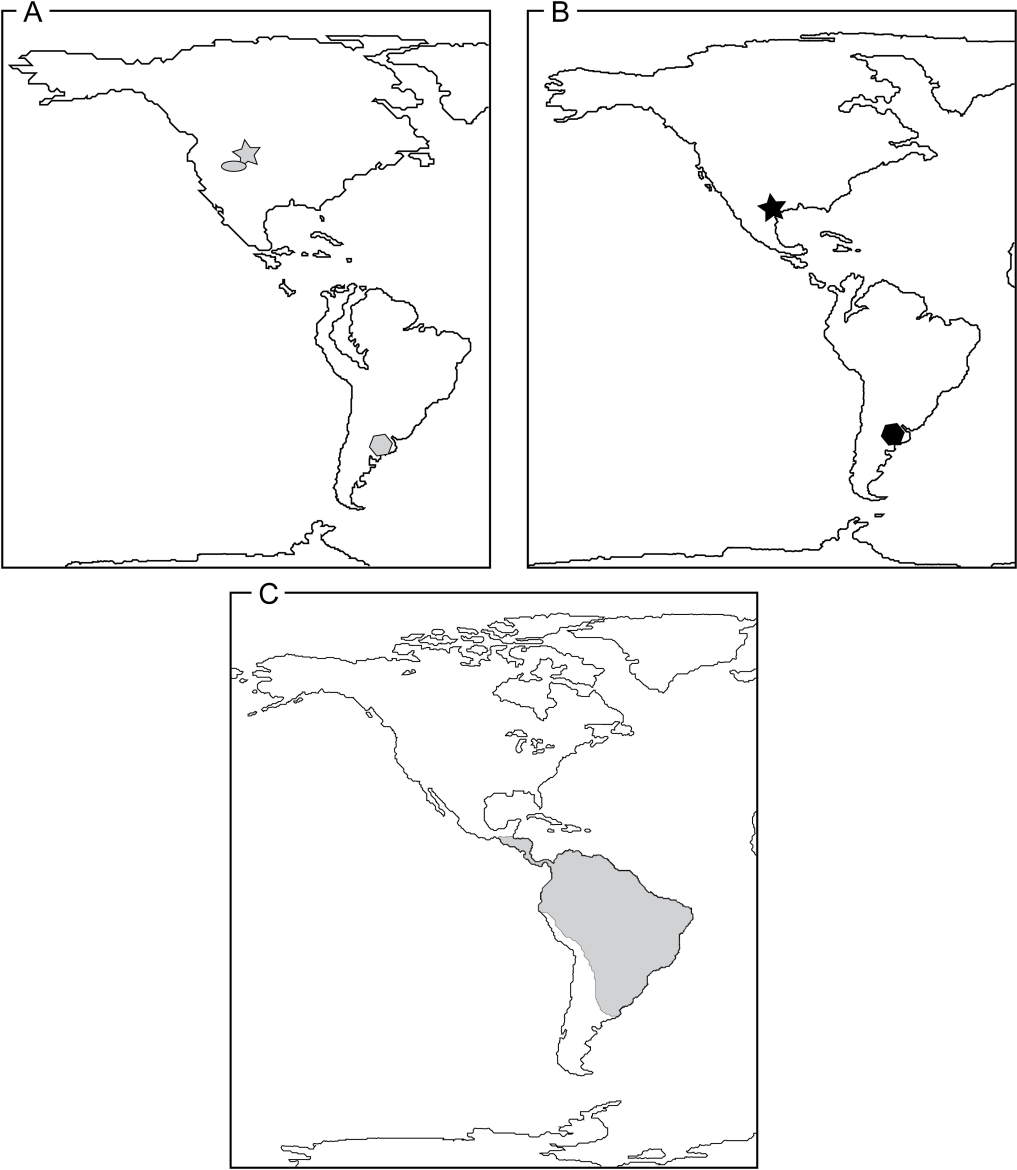
Reconstructions of continental arrangements in the Western Hemisphere depicting known Caimaninae distribution through time. (A) Late Paleocene–Early Eocene. Gray star and gray oval represent *Tsoabichi greenriverensis* and *Orthogenysuchus olseni* from the Wasatchian of Wyoming, USA, and gray polygon represents *Necrosuchus ionensis*from the Paleocene of Chubut Province, Argentina. (B) Middle Eocene–Early Oligocene. Black star represents *Chinatichampsus wilsonorum* from the late Uintan–Duchesnean of Southwest Texas, USA, and black polygon represents *Eocaiman cavernensis* from the Barrancan (Casamayoran) of Chubut Province, Argentina. (C) Present day. Gray area indicates current distribution of Caimaninae.

The biogeographic distribution of extinct alligatorids is unclear (Xu & Huang, 1984; Snyder, 2007). The available fossil evidence suggests that there was a much wider distribution of Alligatoridae in the Paleogene, with the extant distribution of Alligatoridae representing only a “relict of the early Tertiary radiation” (Sill, 1968:76). Molecular estimates for the alligatorine-caimanine divergence are near the Cretaceous-Paleogene Boundary (Roos, Aggarawal & Janke, 2007; Oaks, 2011), but the earliest confirmed skeletal records of alligatorids from the early Paleogene already encompass a broad geographic distribution. The earliest confirmed alligatorine is *Navajosuchus novomexicanus* from the Paleocene of New Mexico (Mook, 1942; Brochu, 2004). Caimanines today occupy a geographic range that extends from southern Mexico to Buenos Aires Province in Argentina (~17°N latitude to ~35°S latitude), excluding introduced populations in Puerto Rico, Cuba, and the United States (Fig. 9). However, their Paleocene and Eocene distribution was much broader (Simpson, 1933, 1937; Brochu, 1999, 2010, 2011; Bona et al., 2018).

Some recent analyses place Late Cretaceous and earliest Paleocene globidontans from North America, such as *Bottosaurus* Agassiz 1849, *Stangerochampsa* Wu et al. 1996 and *Brachychampsa* Gilmore 1911, at the root of Caimaninae, but whether this reflects accurate phylogenetic relationships or a broader phylogenetic distribution of characters currently thought to diagnose Caimaninae among globidontans is unclear (Cossette & Brochu, 2018; Bona et al., 2018; Cidade, Fortier & Hsiou, 2020). The earliest unambiguous caimanines are *Necrosuchus ionensis* Simpson, 1937 (Brochu, 2011; Cidade, Fortier & Hsiou, 2020), *Eocaiman palaeocenicus* Bona, 2007, and *Protocaiman peligrensis* Bona et al., 2018. *Notocaiman stromeri* Rusconi 1937 is slightly younger. All are from the Paleocene of Argentina. Caimanines may have arisen in the Late Cretaceous, but unquestioned material post-dates the K–Pg boundary.

In North America, the caimanine *Tsoabichi greenriverensis* is known from the Wasatchian of Wyoming (Brochu, 2010). *Orthogenysuchus olseni* Mook, 1924, also from the Wasatchian of Wyoming, was recovered as a caimanine related to *Mourasuchus* in some analyses (Brochu, 1999), but further work with the type specimen calls this interpretation into question (Salas-Gismondi et al., 2015, and C. Brochu, 2015, personal observation). Other Paleogene alligatorids from southern California, Montana, Oregon, and Saskatchewan (Repenning & Vedder, 1961; Bryant, 1989; Hanson, 1996; Brochu, 2003, 2004) require reevaluation for more specific identifications, and their re-evaluation may eventually recover additional North American caimanines. Though there are older records of caimanines in both North and South America, *Chinatichampsus wilsonorum* represents the youngest confirmed Paleogene North American caimanine.

Alligatoroid historical biogeography appears to have been far more multifaceted than previously appreciated. At least three lineages—Diplocynodontinae and those including *Hassiacosuchus* and *Arambourgia*—dispersed to Europe independently (Brochu, 1999, 2004). There also appears to have been an early-diverging globidontan clade in the Paleogene of China and Vietnam unrelated to Miocene through Recent Asian *Alligator* (Martin & Lauprasert, 2010; Skutschas et al., 2014; Wang, Sullivan & Liu, 2016; Massonne et al., 2019). Alligatoroidea appears to be an ancestrally North American clade, but it spread beyond the continent multiple times. The complexity of caimanine historical biogeography is confounding because of the lack of a continuous land bridge for most of the Cenozoic, but not unique in the broader context of fossil alligatoroid distributions.

Southern shifts in geographic ranges were proposed for multiple taxa (e.g., Chelydridae, Dermatemyidae, Boinae, Primates, Strisores) in the later portion of the Eocene (Matthew, 1939; Estes, 1970; Estes & Hutchison, 1980; Markwick, 1998; Holman, 2000; Williams & Kirk, 2008; Kirk & Williams, 2011; Nesbitt, Ksepka & Clarke, 2011). Those hypothesized shifts supported the idea that changes in the climatic conditions in the central Rocky Mountains from warm and humid forests to cooler and seasonally drier forests through the Eocene caused taxa to take refuge in the warm and humid environment that was interpreted for southern North America later in the Eocene. Paleoclimatic reconstructions for the Paleocene–Eocene Thermal Maximum (PETM) in the Bighorn Basin placed the mean annual temperature (MAT) at ~55 Ma at approximately 20 °C using leaf margin analysis as the proxy (Wing et al., 2005). Other estimates derived from carbonate clumped isotope thermometry of paleosol carbonates estimated the MAT in the Bighorn Basin much higher at ~38 °C at the Paleocene-Eocene boundary (Snell et al., 2013). The Early Eocene Climatic Optimum (EECO; 53–51 Ma) comprised the warmest interval, with the highest atmospheric carbon dioxide estimates for the entire Eocene (Zachos et al., 2001). A substantially cooler MAT was estimated at ~15 °C later at the Eocene–Oligocene boundary in the northern Great Plains (Zanazzi et al., 2007).

Mean annual temperature estimations for the early middle Eocene of Texas, based on stable isotope profiles of shallow water gastropods, were 27–28 °C with a seasonal range of 8–9 °C (Andreasson & Schmitz, 2000). The middle and late Eocene conditions in West Texas were inferred to be a continuation of warm and humid climates based on the reported occurrence of *Nypa* palm seeds from the Casa Blanca local fauna (Westgate & Gee, 1990) and the known occurrence of primate taxa (Williams & Kirk, 2008; Kirk & Williams, 2011). Both primates and *Nypa* palms have mainly tropical distributions today, and a tropical paleoecology was inferred for their Eocene representatives.

The observed distribution of fossils of Caimaninae could be interpreted as a pattern of range contraction for the clade as it tracked higher temperatures and humidity that exist today in the Amazon Basin and southern Central America (Sill, 1968; Steel, 1973; Brochu, 1999, 2003, 2011). In this scenario, we see multiple caimanines in higher latitude North American fossil assemblages early in the Eocene, but later they are only found at lower latitudes (DGF in the latest Uintan). The extirpation of caimanine alligatorids by the Chadronian resulted in depauperate faunal assemblages in North America, in which the only known crocodyliform is *Alligator prenasalis*. However, this could be an overinterpretation using a poor fossil record (Brochu, 2010, 2011), and recovery of specimens of caimanines from the Paleogene of Central America and additional specimens from the Paleogene of South America would add important biogeographic data to this issue. The new record from the late Uintan–Duchesnean portion of the Devil’s Graveyard Formation begins to bridge that crucial chronologic and geographic gap between the North American records in the early Eocene and the modern, Central and South American distribution of caimanines.

## Institutional Abbreviations

AMNH: American Museum of Natural History, New York, New York, USA
SDSM: South Dakota School of Mines and Technology, Rapid City, South Dakota, USA
TMM: Texas Vertebrate Paleontology Collections, Jackson School Museum of Earth History, Austin, Texas, USA
YPM: Yale Peabody Museum, New Haven, Connecticut, USA.

## Additional Information and Declarations

### Field Study Permissions

The following information was supplied relating to field study approvals (i.e., approving body and any reference numbers): Fieldwork was approved by Midwestern State University.

### Data Availability

The following information was supplied regarding data availability: The matrix and character list are available on MorphoBank under Project ID 2781 (https://morphobank.org/index.php/Projects/ProjectOverview/project_id/2781). TMM 45911-1 is accessioned in the Texas Vertebrate Paleontology Collections of The University of Texas at Austin, Austin, Texas, USA.

### New Species Registration

The following information was supplied regarding the registration of a newly described species: Publication LSID: urn:lsid:zoobank.org:pub:00150C1B-9E7D-45CE-8648-12CF2188310C *Chinatichampsus* gen. nov. LSID: urn:lsid:zoobank.org:act:91246A7E-C9EA-4206-8A87-6205E3684989 *Chinatichampsus wilsonorum* gen. et sp. nov. LSID: urn:lsid:zoobank.org:act:9BA8A50C-3A37-40B2-9B35-B4D853A18890.
